# Purification and characterization of a thermophilic NAD ^+^‐dependent lactate dehydrogenase from *Moorella thermoacetica*

**DOI:** 10.1002/2211-5463.13964

**Published:** 2025-01-13

**Authors:** Florian P. Rosenbaum, Volker Müller

**Affiliations:** ^1^ Department of Molecular Microbiology & Bioenergetics, Institute of Molecular Biosciences Johann Wolfgang Goethe University Frankfurt am Main Germany

**Keywords:** acetogen, anaerobe, lactate, purification, thermophile

## Abstract

Oxidation of lactate under anaerobic dark fermentative conditions poses an energetic problem. The redox potential of the lactate/pyruvate couple is too electropositive to reduce the physiological electron carriers NAD(P)^+^ or ferredoxin. However, the thermophilic, anaerobic, and acetogenic model organism *Moorella thermoacetica* can grow on lactate but was suggested to have a NAD^+^‐dependent lactate dehydrogenase (LDH), based on enzyme assays in cell‐free extract. LDHs of thermophilic and anaerobic bacteria are barely characterized but have a huge biotechnological potential. Here, we have purified the LDH from *M. thermoacetica* by classical chromatography. Lactate‐dependent NAD^+^ reduction was observed with high rates. Electron bifurcation was not observed. At pH 8 and 65 °C, the LDH had a specific activity of 60 U·mg^−1^ for lactate oxidation, but NADH‐driven pyruvate reduction was around four times faster with an activity of 237 U·mg^−1^. Since lactate formation is preferred by the enzyme, further modifications of the LDH can be suggested to improve the kinetics of this enzyme making it a promising candidate for biotechnological applications.

## Graphical abstract

The thermophilic acetogenic model organism *Moorella thermoacetica* can utilize lactate as substrate. The formation of lactate by reduction of pyruvate is energetically favorable, but the reverse reaction, the oxidation of lactate to pyruvate with NAD^+^ as electron acceptor is challenging. To shed light into enzymology of the lactate metabolism in *M. thermoacetica* we purified and characterized the NAD^+^‐dependent l‐lactate dehydrogenase.

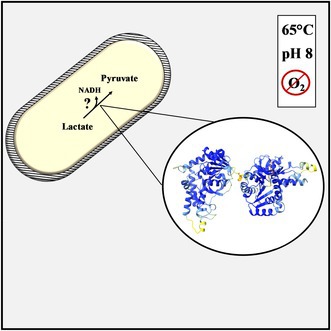

**Abbreviations**
AQDS: anthraquinone‐2,6‐disulfonate
BV: benzyl viologen
CE: cell‐free extract
CODH/ACS: carbon monoxide dehydrogenase/acetyl‐CoA synthase
CP: cytoplasm
DCPIP: 2,6‐dichlorophenolindophenol
ETF: electron transfer flavoprotein
FBP: fructose‐1,6‐bisphosphate
Fd: ferredoxin
LDH: lactate dehydrogenase
MQ: menaquinone
MQH_2_
: menaquinol
MV: methyl viologen
THF: tetrahydrofolate
WLP: Wood–Ljungdahl pathway

Acetogenic bacteria are a phylogenetically diverse group of anaerobic bacteria that utilize a broad range of substrates including sugars, alcohols, or carbonic acids or gaseous substrates such as H_2_ plus CO_2_ or CO [1]. Oxidation of the substrates is coupled to the reduction of the soluble electron carriers NADP^+^ (*E*′ = −370 mV), NAD^+^ (*E*′ = −280 mV), or ferredoxin (*E*_0_′ ≈ −450 mV) that are re‐oxidized by reducing CO_2_ to acetate in the Wood–Ljungdahl pathway (WLP, Fig. 1) [2, 3, 4]. Lactate is a substrate for many acetogens and oxidized by a lactate dehydrogenase to pyruvate which is converted by pyruvate:ferredoxin oxidoreductase to acetyl‐CoA, CO_2_, and reduced ferredoxin; acetyl‐CoA is further converted to acetate with the concomitant synthesis of 1 mol of ATP by substrate‐level phosphorylation in the acetate kinase reaction [5, 6, 7]. However, lactate oxidation causes a thermodynamic problem, the redox potential (*E*_0_′) of the lactate/pyruvate pair is rather electropositive with −190 mV; therefore, lactate oxidation cannot be directly coupled to any of the before‐mentioned electron carriers [8]. This thermodynamic problem is solved in the non‐acetogen *Escherichia coli* by using ubiquinone (*E*_0_′ = +113 mV) as electron acceptor. The acetogen *Acetobacterium woodii* uses a different strategy: endergonic NAD^+^ reduction occurs but is driven by the simultaneous oxidation of reduced ferredoxin, and both reactions are coupled in one soluble, electron‐bifurcating lactate dehydrogenase (LDH)/electron transfer flavoprotein (ETF) complex [5, 9].

**Fig. 1:**
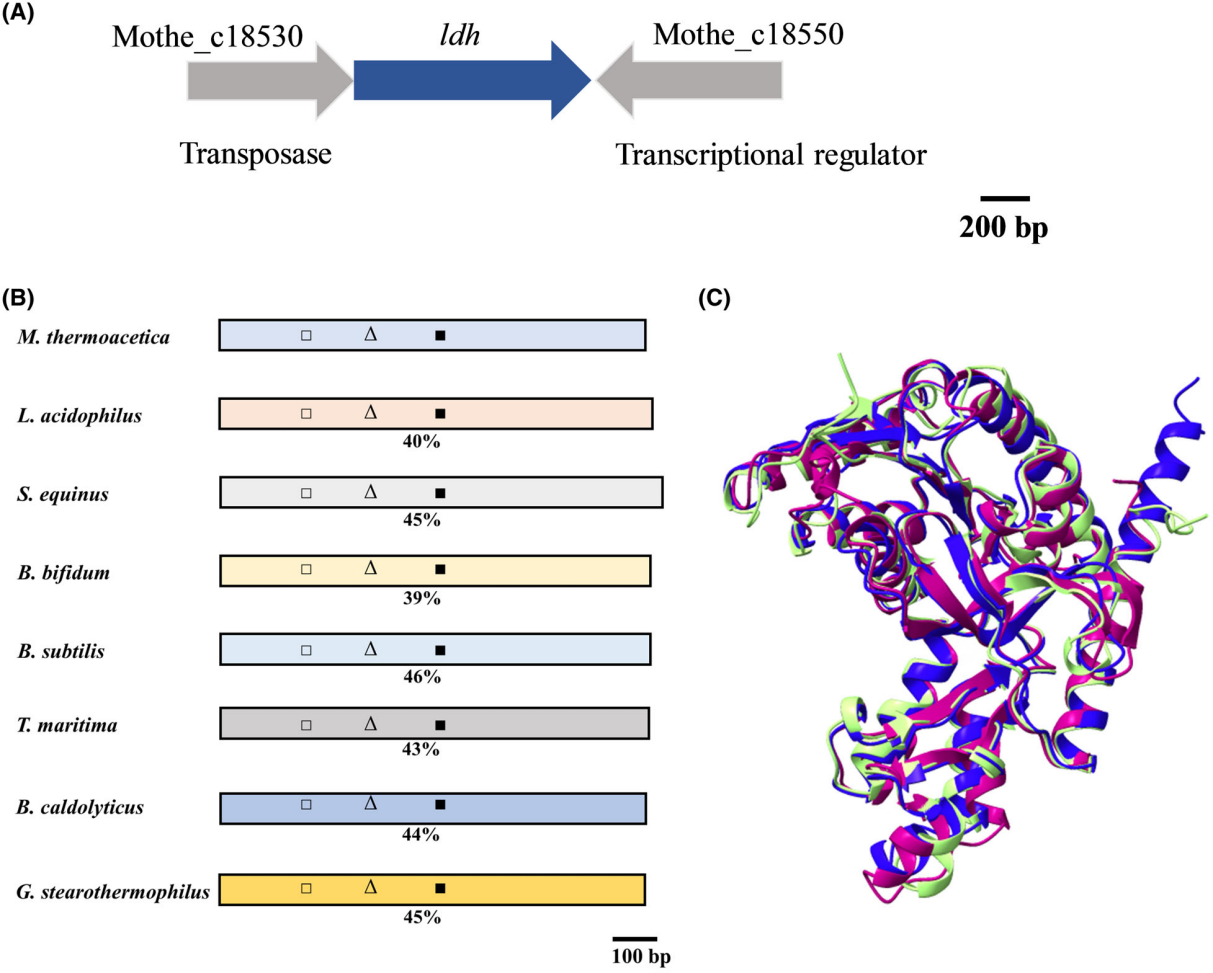
Genetic organization and comparison of the LDH from *Moorella thermoacetica* to LDHs from other bacteria. The potential LDH gene is colored in blue. The LDH gene is surrounded by the genes Mothe_c18530 and Mothe_c18550, encoding for a putative transposase and a transcriptional regulator (A). The LDH was compared to the LDH from *Lactobacillus acidophilus*, *Streptococcus equinus*, *Bifidobacterium bifidum*, *Bacillus subtilis*, *Thermotoga maritima*, *Bacillus caldolyticus*, and *Geobacillus stearothermophilus*. The identity is given in percent. □, Rossmann fold NAD(P)^+^ binding domain; Δ, fructose‐1,6‐bisphosphate‐binding domain; ■, active site (B). A structural overlay of the LDH from *M. thermoacetica* (purple), *T. maritima* (blue), and *B. subtilis* (yellow) indicates structural similarity (C).

Yet, the thermophilic acetogen *Moorella thermoacetica* seems to have a different way to overcome the energetic hurdle. Measurements in cell‐free extracts revealed a lactate‐dependent NAD^+^ reduction and transcriptome analysis revealed one gene encoding a potential LDH to be upregulated [6]. Both findings were interpreted as evidence for a NAD^+^‐dependent LDH in *M. thermoacetica*. However, these measurements in cell‐free extracts do neither exclude an electron‐bifurcating LDH that simultaneously uses lactate and reduced ferredoxin as electron donors nor the involvement of other electron carriers in the process. Therefore, the mechanism of lactate oxidation remained elusive.

Here, we report the purification of a thermophilic, single‐subunit LDH from *M. thermoacetica* whose predicted structure is similar to the one of the NAD^+^‐specific LDH from *Bacillus subtilis*. The enzyme uses NAD^+^ as electron acceptor, electron confurcation with ferredoxin was not observed. Of interest for biotechnological applications is the 4 times faster formation of lactate from pyruvate with NADH as electron donor. Lactate is the precursor for polylactic acid, the bioplastic with the highest consumption volume of any bioplastics and the enzyme could be used to produce lactate under thermophilic condition from renewable feedstocks [10].

## Methods

### Growth conditions

*Moorella thermoacetica* (DSM 521) was cultivated under anoxic conditions at 55 °C in bicarbonate‐buffered complex medium as previously described [6]. The medium was prepared using the anaerobic techniques described previously [11, 12]. Cells were grown in 1‐ or 20‐L‐flasks (Glasgerätebau Ochs, Bovenden/Lenglern, Germany) supplemented with 80 mm d/l‐lactate as carbon and energy source. d/l‐lactate was prepared by neutralization of d/l‐lactic acid with NaOH.

### Purification of the LDH

*Moorella thermoacetica* was cultivated as described above, and all following steps were carried out in an anaerobic chamber (Coy Laboratories, Grass Lake, MI, USA) containing an N_2_ + H_2_ (95 + 5 [v/v]) atmosphere. Cultures were harvested and washed twice with harvest buffer (50 mm Tris/HCl (pH 7.5), 20 mm MgSO_4_ × 7 H_2_O, 20% glycerol, 4 mm DTE, 4 μm resazurin). Cell‐free extract, cytoplasm, and membranes were prepared as described [6]. For further enzyme purification, only cytoplasm was used. The cytoplasm was applied to a Q‐ Sepharose FF column (GE Healthcare, Chicago, IL, USA) equilibrated with buffer 1 (50 mm Tris/HCl (pH 7.5), 20 mm MgSO_4_ × 7 H_2_O, 20% glycerol, 4 mm DTE, 4 μm resazurin) with a flow rate of 2 mL·min^−1^. The proteins were eluted from the column with a linear gradient of 200 mL from 0 to 1 m NaCl in buffer 2 (50 mm Tris/HCl (pH 7.5), 20 mm MgSO_4_ × 7 H_2_O, 20% glycerol, 4 mm DTE, 4 μm resazurin, 1 m NaCl) using a flow rate of 2 mL·min^−1^. Fractions having lactate:NAD^+^ oxidoreductase activity were pooled for further purification. Afterwards, 0.86 m (NH_4_)_2_SO_4_ was added to the pooled fractions and proteins were loaded with 2 mL·min^−1^ on a Phenyl‐Sepharose HP column (GE Healthcare) equilibrated with buffer 3 (50 mm Tris/HCl (pH 7.5), 0.86 m (NH_4_)_2_SO_4_, 20 mm MgSO_4_ × 7 H_2_O, 20% glycerol, 4 mm DTE, 4 μm resazurin). Proteins were eluted from the column using a linear gradient of 200 mL from 0.86 to 0 m (NH_4_)_2_SO_4_ with a flow rate of 2 mL·min^−1^. After proteins were eluted from the Phenyl‐Sepharose HP column, buffer 3 was exchanged to H_2_O. Proteins eluted from the column were fractioned and tested for lactate‐dependent NAD^+^ reduction. Fractions having lactate:NAD^+^ oxidoreductase activity were pooled for further purification. Pooled fractions were concentrated by ultrafiltration using 30‐kDa Vivaspin tubes (Sartorius Stedim Biotech GmbH, Göttingen, Germany). Afterwards, the sample was loaded on a Superose 6 increase 10/300 GL prepacked column (GE Healthcare, Chicago, IL, USA) equilibrated with buffer 4 (50 mm Tris/HCl (pH 7.5), 150 mm NaCl, 20 mm MgSO_4_ × 7 H_2_O, 20% glycerol, 4 mm DTE, 4 μm resazurin). Size exclusion chromatography was performed with a rate of 0.3 mL·min^−1^. Fractions having lactate:NAD^+^ oxidoreductase activity were pooled and stored at 4 °C.

### Measurement of LDH activity

All enzyme assays were carried out at 65 °C in 1.8 mL anoxic cuvettes (Glasgerätebau Ochs) filled with enzyme buffer A (50 mm Tris/HCl (pH 8), 10 mm NaCl, 4 mm DTE, 4 μm resazurin) at a final volume of 1 mL under a 100% N_2_ gas atmosphere. The pH dependence of the lactate:NAD^+^ reaction was measured in buffer B1 (25 mm Tris/HCl, 25 mm CHES, 25 mm MES, 25 mm MOPS (pH 6–10), 10 mm NaCl, 4 mm DTE, 4 μm resazurin). The pH dependence of the pyruvate:NADH reaction was measured in buffer B2 (25 mm Tris, 25 mm CHES, 25 mm MES, 25 mm MOPS, 25 mm citrate (pH 4–9), 10 mm NaCl, 4 mm DTE, 4 μm resazurin). The *K*_m_ was determined using buffer C (50 mm Tris/HCl (pH 8), 10 mm NaCl, 4 mm DTE, 4 μm resazurin). LDH activity was measured with 1 mm methyl viologen at 604 nm (ε methyl viologen = 13.9 mm ^−1^·cm^−1^), 1 mm benzyl viologen at 600 nm (ε benzyl viologen = 12 mm ^−1^·cm^−1^), 50 μm DCPIP at 600 nm (ε DCPIP = 20.7 mm ^−1^·cm^−1^), 1 mm K_3_Fe(CN)_6_ 420 nm (ε K_3_Fe(CN)_6_ = 1 mm ^−1^·cm^−1^), 0.25 mm AQDS at 408 nm (ε AQDS = 7.8 mm ^−1^·cm^−1^), 30 μm Fd at 430 nm (ε Fd = 13.1 mm ^−1^·cm^−1^), or 0–2 mm NAD(P)^+^/H at 340 nm (ε NAD(P)^+^/H = 6.2 mm ^−1^·cm^−1^). One unit is defined as transfer of 2 μmol electrons·min^−1^. Enzyme activity assays were conducted using 0–5 mm pyruvate, 0–5 mm phosphoenolpyruvate, 0–100 mm l‐lactate, 20 mm d‐lactate, dl‐malate, malonate, glycolate, thioglycolate, oxalate, formate, fumarate, succinate, propionate, or butyrate. In addition, enzyme activity assays were conducted using 1 mm fructose‐1,6‐bisphosphate and 5 mm pyruvate or 20 mm l‐lactate.

### Analytical methods

The concentration of proteins was measured according to Bradford [13]. Proteins were separated in 12% polyacrylamide gels and stained with Coomassie brilliant blue G250 [14]. The molecular mass of the purified LDH was determined using a calibrated superose 6 column, buffer 4 and defined size standards (aprotinin: 6.5 kDa; carbonic anhydrase: 29 kDa; ovalbumin: 44 kDa; conalbumin: 75 kDa). The isolated LDH was identified by MALDI‐TOF analysis. Peptide mass fingerprinting was performed by the ‘Functional Genomics Center Zürich’ at the ETH Zurich, Switzerland, and results were analyzed using the scaffold‐proteome software version 5.3.0 (Proteome Software Inc., Portland, OR, USA). Ferredoxin was isolated from *Clostridium pasteurianum* and *Thermoanaerobacter kivui* (TKV_c16450) [3, 15].

### Prediction of the molecular architecture

Modeling of the LDH was performed using AlphaFold2 via the ColabFold pipeline applying mostly default parameters (use_amber: no, template mode: none, msa_mode: MMSeq2 (UniRef and Environmental), num_recycle: 3). The resulting predicted structures with the highest model confidence (based on pLDDT and predicted aligned error (PAE) confidence measures) [16, 17]. Structure was analyzed using chimerax [18].

## Results

### Genetic organization of the LDH from *M. thermoacetica* and comparison to other LDHs

The LDH encoding gene, *ldh*, is 954 bp long. A putative ribosomal binding region is found 10 bp in front (gggagg) and a transcriptional terminator is 14 bp downstream of the *ldh* gene. ATG is used as a start codon and TAG as a stop codon. Upstream of the *ldh* gene is a gene encoding a potential transposase and downstream in the opposite direction is *sinR2*, encoding a potential transcriptional regulator (Fig. 1A). The predicted LDH has a molecular mass of 34.6 kDa and is 40%, 45%, 39%, 46%, 43%, 44%, and 45% identical to the NAD^+^‐dependent LDH of *Lactobacillus acidophilus*, *Streptococcus equinus*, *Bifidobacterium bifidum*, *B. subtilis*, *Thermotoga maritima*, *Bacillus caldolyticus*, and *Geobacillus stearothermophilus*, respectively (Fig. 1B). The predicted molecular mass of these proteins is similar with masses ranging from 34.1 to 35.6 kDa. The LDH from *M. thermoacetica* has a predicted binding site for NAD(P)^+^ (binding coordinated by amino acids V17, D38, K43, G82, A83, V121, A121, N123, and S146). The substrate binding domain is predicted to be coordinated by the amino acids Q85, R91, P126, V127, D128, D151, S152, A153, R154, and T233. Those residues are mostly conserved among the enzymes used as reference (Fig. S1). The NAD(P)^+^‐dependent LDHs can be grouped into allosterically and nonallosterically regulated LDHs. Allosteric LDHs are regulated by fructose‐1,6‐bisphosphate (FBP), and a FBP‐binding site is also present in the enzyme from *M. thermoacetica*, coordinated by the amino acids R156 and H171 as well as in the NAD^+^‐dependent LDHs from *L. acidophilus*, *S. equinus*, *B. bifidum*, *B. subtilis*, *T. maritima*, *B. caldolyticus*, and *G. stearothermophilus*. AlphaFold2 models of the enzymes from *B. subtilis M. thermoacetica* and *T. maritima* are very similar (Fig. 1C).

### Purification of the LDH from *M. thermoacetica*

**Fig. 2:**
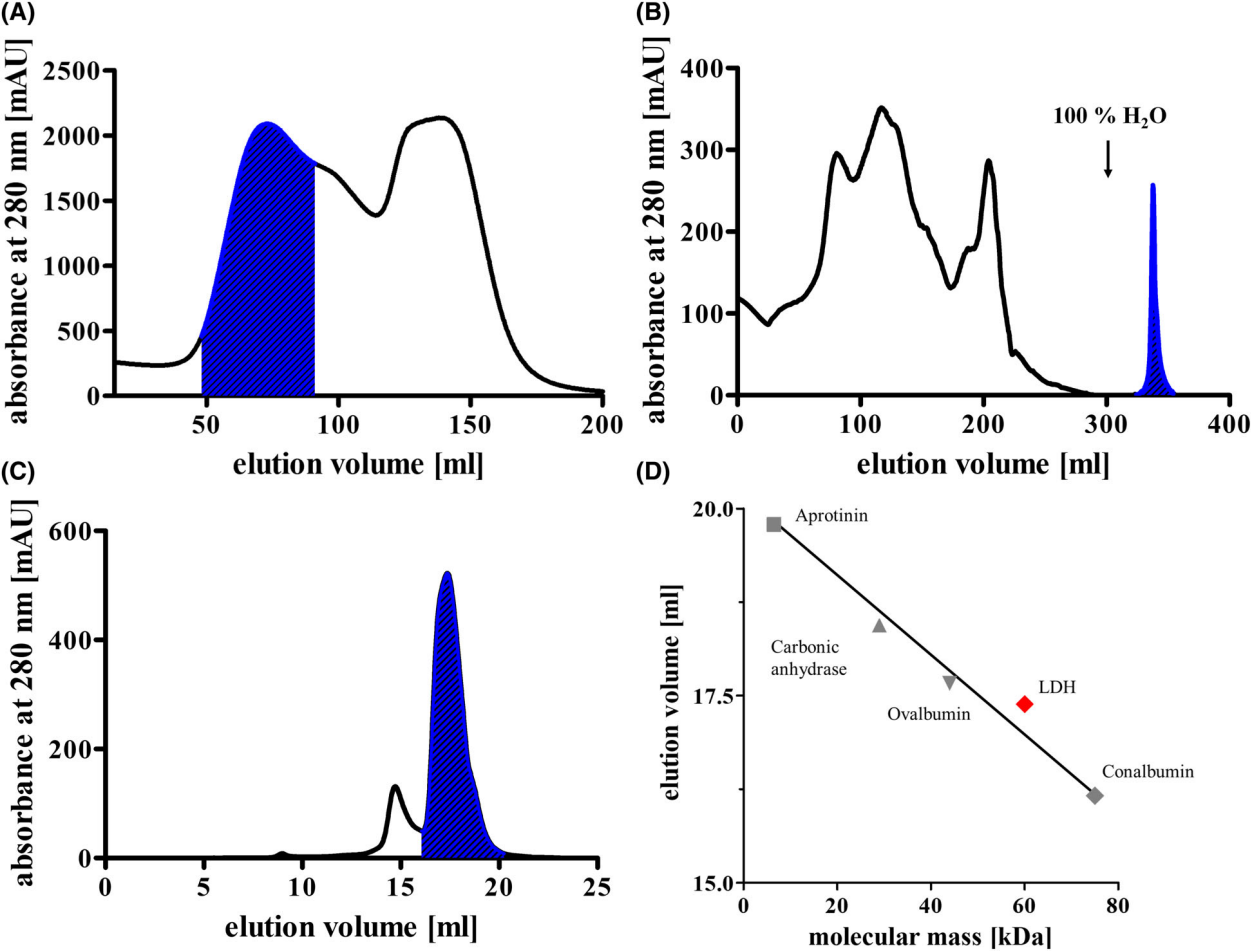
Purification of the LDH from *Moorella thermoacetica*. Proteins of the cytoplasm were separated by anion exchange chromatography using a NaCl gradient from 0 to 1 m NaCl over 200 mL. Fractions containing LDH activity are indicated by blue shading (A). Pooled fraction after separation by anion exchange chromatography were precipitated and further separated by hydrophobic interaction chromatography. Fractions containing LDH activity are indicated by blue shading (B). Pooled fraction after hydrophobic interaction chromatography were concentrated by ultrafiltration and further separated by size exclusion chromatography. The LDH eluted in a single peak (C, blue shading). Size exclusion chromatography column was calibrated using aprotinin (6.5 kDa), carbonic anhydrase (29 kDa), ovalbumin (44 kDa), and conalbumin (75 kDa) as standard proteins, indicated by gray symbols (D). The elution behavior of the LDH is indicated by the red symbol.

**Fig. 3:**
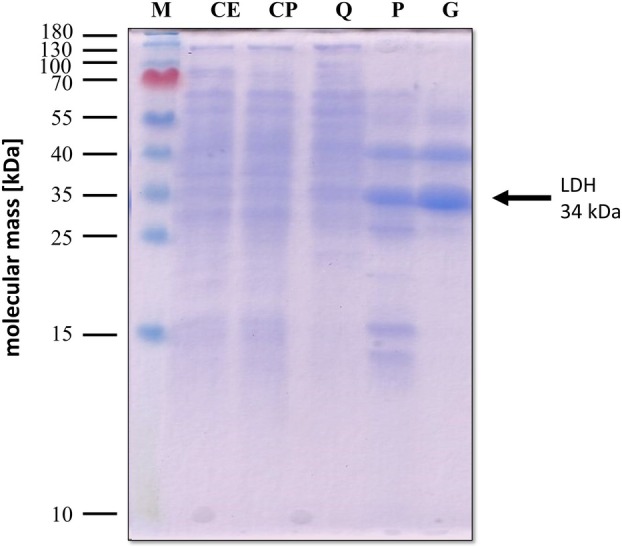
SDS/PAGE monitoring the purification process of the LDH from *Moorella thermoacetica*. Samples of the different purification steps were separated by SDS/PAGE (12%), and proteins were stained with Coomassie Brilliant Blue G250. Ten microgram of protein was applied to each lane. CE, cell‐free extract; CP, cytoplasm; M, prestained page ruler; P, pooled fractions from Phenyl‐ Sepharose; Q, pooled fractions from Q‐ Sepharose; S, pooled fractions from size exclusion Superose 6.

Cell‐free extract of lactate‐grown cells had a LDH activity of 0.64 U·mg^−1^ of protein using lactate as electron donor and NAD^+^ as electron acceptor, glucose‐grown cells had a two orders of magnitude lower activity of around 2 mU·mg^−1^. After separating soluble proteins and membranes, the activity increased to 0.71 U·mg^−1^ in the soluble fraction. The proteins of the cytoplasm were separated by anion exchange chromatography on Q‐Sepharose, and the LDH eluted at a NaCl concentration of 30–130 mm (Fig. 2A). Fractions with highest activity were pooled, precipitated with 20% ammonium sulfate, and further separated by hydrophobic interaction chromatography on Phenyl‐Sepharose. Interestingly, the LDH had a very high affinity to the column matrix and eluted only, when water was used as solvent (Fig. 2B). Fractions showing the highest activity were again pooled, concentrated using a 30‐kDa Vivaspin ultrafiltration tube, and separated by size exclusion chromatography; the LDH eluted in a single peak at 17.39 mL (Fig. 2C). Using this procedure, the enzyme was enriched 94‐fold with a specific activity of 60.2 U·mg^−1^ under optimal conditions (65 °C, pH 8.0, 100 mm lactate) and a yield of 16% (Table 1). The native size of the LDH was 60 kDa, as determined by size exclusion chromatography (Fig. 2D). In order to monitor the progress of the purification and the purity of the protein, samples from each purification step were applied to a denaturing SDS gel, showing one dominant protein of approximately 34 kDa, which fits to the predicted size of the monomeric LDH (Fig. 3). The 34‐kDa protein was identified as the gene product of the *ldh* gene by peptide mass fingerprinting. In addition, the co‐eluting protein with a molecular mass of 40 kDa was identified as an aminotransferase (Mothe_c00180).

**Table 1:** Purification of the LDH from *Moorella thermoacetica.*

Purification step	Protein (mg)	Volume (mL)	LDH activity (U)	LDH activity (U·mL^−1^)	LDH activity (U·mg^−1^)	Purification (‐fold)	Yield (%)
Cell‐free extract	1918.9	31	1228.09	39.44	0.64	1	100
Cytoplasm	1638.8	34.5	1163.55	33.95	0.71	1.11	95
Q‐ Sepharose	433.4	48	710.78	14.79	1.64	2.56	58
Phenyl‐ Sepharose	8.7	15	438.21	29.22	50.37	78.70	36
Superose 6	3.2	2.5	192.58	77.03	60.18	94.03	16

### Basic biochemical properties of the LDH from *M. thermoacetica*

**Fig. 4:**
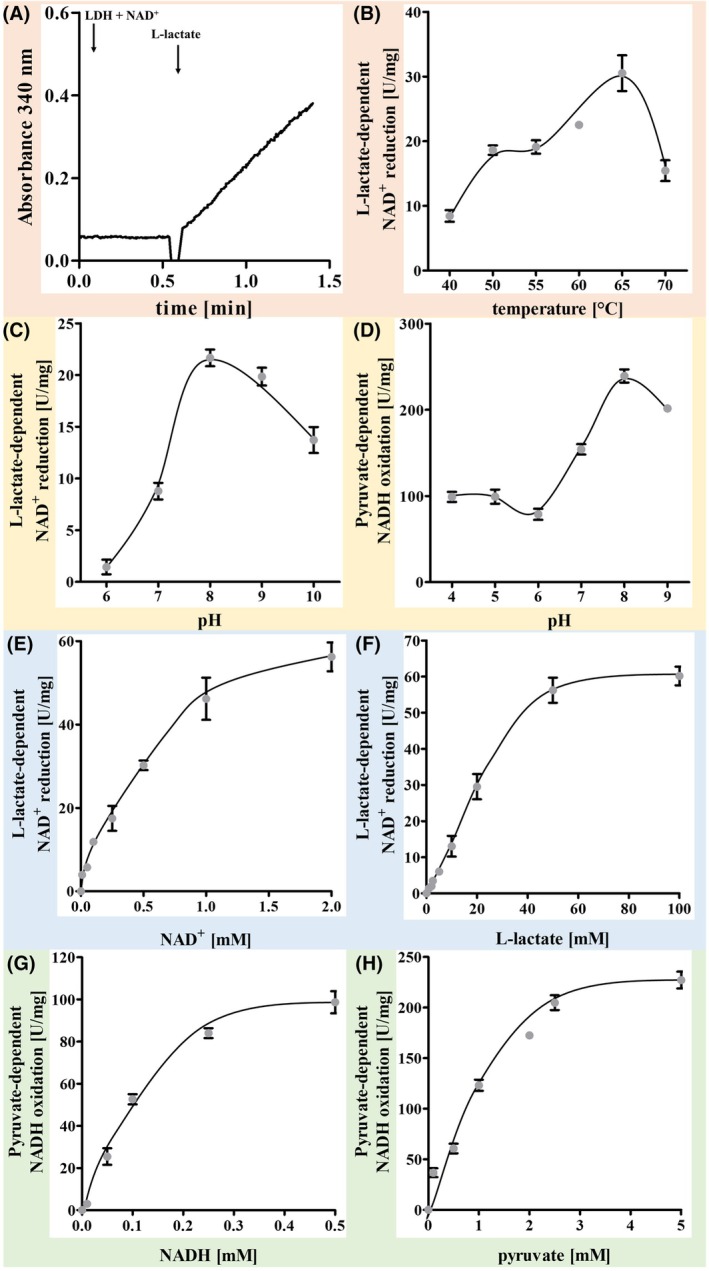
Biochemical characterization of the LDH. All assays were performed in 1.8‐mL anoxic cuvettes containing an overall liquid volume of 1 mL and 5 μg protein and a 100% N_2_ atmosphere. (A) lactate‐dependent NAD^+^ reduction. The assay contained 2 mm NAD^+^ in buffer A. (B) Temperature optimum of the LDH, the assay contained 2 mm NAD^+^ in buffer A at 40–70 °C. (C) pH optimum of the lactate‐dependent NAD^+^ reduction, the assay contained 2 mm NAD^+^ in buffer B1 at 65 °C. (D) Pyruvate‐dependent NADH oxidation of the LDH, the assay contained 2 mm NADH in buffer B2 at 65 °C. (E) *K*_m_ determination for NAD^+^, and the assay contained 0–2 mm NAD^+^ in buffer A at 65 °C. (F) *K*_m_ determination for l‐lactate. The assay contained 2 mm NAD^+^ and 0–100 mm l‐lactate in buffer A at 65 °C. (G) *K*_m_ determination for NADH. The assay contained 0–0.5 mm NADH and 1 mm pyruvate in buffer A at 65 °C. (H) *K*_m_ determination for pyruvate. The assay contained 0.5 mm NADH and 0–5 mm pyruvate in buffer A at 65 °C. The reactions were started by adding 20 mm l‐lactate or 5 mm pyruvate, if not otherwise stated (*n* = 3; SD).

Next, we determined key biochemical properties of the purified LDH. The enzyme reduced NAD^+^ and with a 100‐fold lower activity also NADP^+^ (0.34 U·mg^−1^ with 20 mm lactate) (Fig. 4A). Ferredoxin (Fd, isolated from *C. pasteurianum* and *T. kivui* (TKV_c16450)) alone or in the presence of NAD^+^ was not reduced. Artificial electron acceptors such as methyl viologen (MV), benzyl viologen (BV), 2,6‐dichlorophenolindophenol (DCPIP), potassium ferricyanide (K_3_Fe(CN)_6_), and the water‐soluble quinone analogue anthraquinone‐2,6‐disulfonate (AQDS) were not reduced. The FBP‐binding site suggests regulation (stimulation) of LDH activity by FBP. However, we could not observe any stimulating (nor inhibitory) effect of FBP on LDH. The LDH only oxidized l‐lactate; d‐lactate, dl‐malate, malonate, glycolate, thioglycolate, oxalate, formate, fumarate, succinate, propionate, and butyrate were not accepted as substrates. Pyruvate was reduced with an activity of 208.85 U·mg^−1^. No activity was observed with phosphoenolpyruvate.

The purified LDH was active between 40 °C (8.4 ± 0.9 U·mg^−1^) and 70 °C (15.4 ± 1.6 U·mg^−1^), and the temperature optimum was at 65 °C (29.5 ± 3.5 U·mg^−1^) (Fig. 4B). The pH range for lactate‐dependent NAD^+^ reduction was from 6 (1.4 ± 0.7 U·mg^−1^) to 10 (13.7 ± 1.3 U·mg^−1^) with an optimum at pH 8 (21.7 ± 0.8 U·mg^−1^) (Fig. 4C). All these measurements were done with 20 mm lactate. Since most LDHs have different pH optima for the forward and backward reaction, we also determined the pH optimum for pyruvate‐dependent NADH oxidation. This activity was observed between pH 4 (100.0 ± 5.8 U·mg^−1^) and pH 9 (201.7 ± 3.6 U·mg^−1^) with an optimum at pH 8 (237.2 ± 8.3 U·mg^−1^) (Fig. 4D). The activity was dependent on the lactate concentration, and optimal activity was observed at 80–100 mm lactate (measured at optimal pH and temperature); the *K*_m_ for lactate was 20 mm. The reaction was also dependent on the NAD^+^ concentration with saturation at ≈ 2 mm and half‐maximal activity at ≈ 0.5 mm. Pyruvate reduction was also observed, optimal activity was at 4–5 mm and half maximal at 1 mm pyruvate. The reaction was also dependent on the NADH concentration with saturation at only 0.5 mm and half‐maximal activity at 0.1 mm (Fig. 4E–H).

## Discussion

**Fig. 5:**
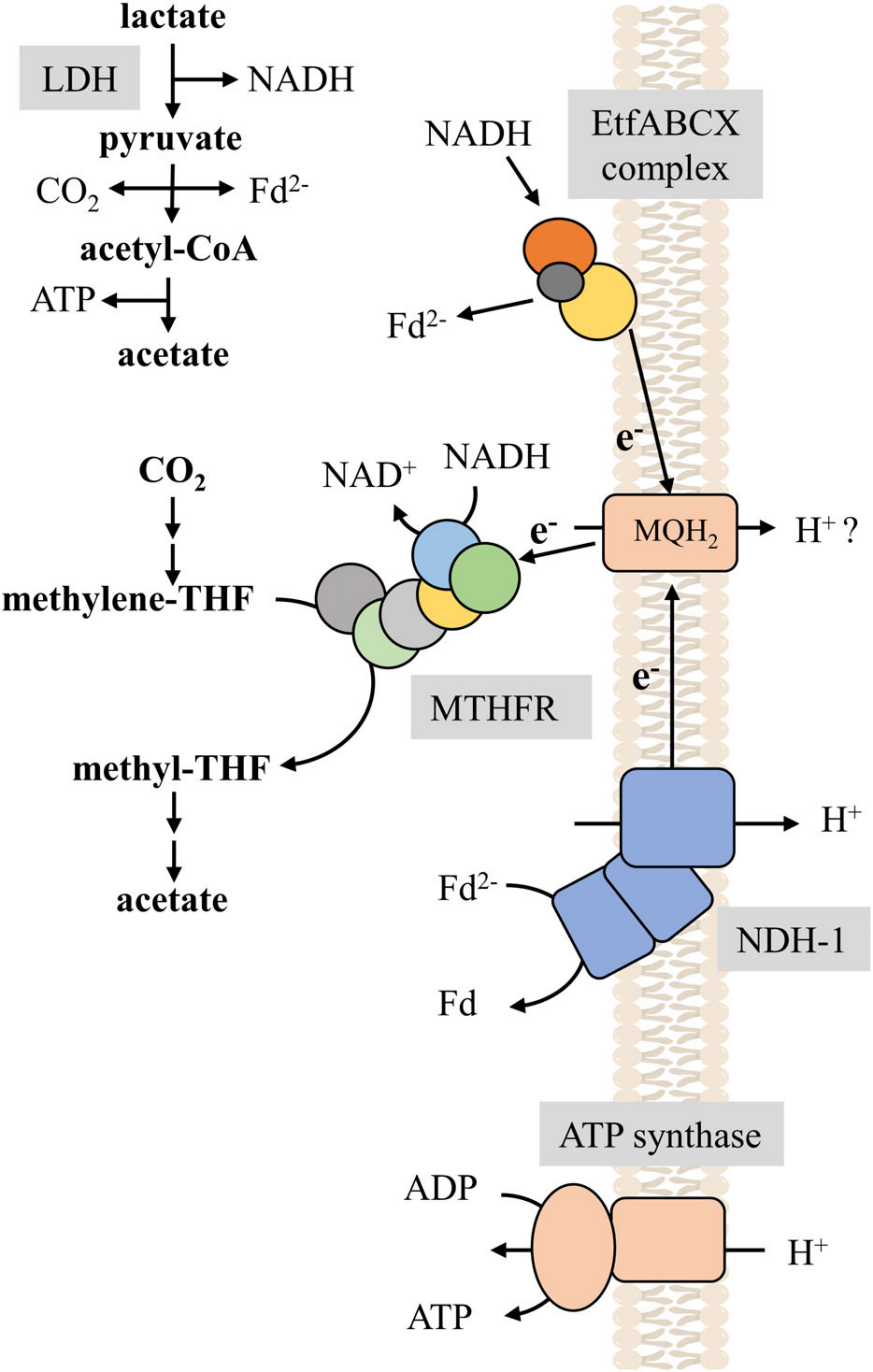
Scheme of lactate metabolism in *Moorella thermoacetica*. It is assumed that the endergonic reduction of NAD^+^ with lactate as electron donor becomes feasible by coupling this reaction to two electron‐bifurcating enzymes [6]. First, the electron‐bifurcating EtfABCX complex oxidizes NADH released by the oxidation of lactate to pyruvate and reduces MQ and Fd. Next, the electron‐bifurcating MTHFR reduces the intermediates methylene‐THF to methyl‐THF of the Wood–Ljungdahl pathway with MQ and NADH as reductants. Methyl‐THF is than further metabolized in the Wood–Ljungdahl pathway to acetate (adapted from Ref. [6]). The reduced Fd is used by ferredoxin‐dependent NADH‐dehydrogenase NDH‐1 to establish the chemiosmotic gradient. This gradient fuels the ATP synthase to generate ATP.

Here we have purified and characterized the LDH from *M. thermoacetica*. This enzyme catalyzes the reversible hydrogenation of pyruvate to lactate with NADH as reductant, allowing to produce and utilize lactate [19]. In contrast to the electron‐bifurcating LDH of *A. woodii*, the LDH form *M. thermoacetica* is indeed a soluble, NAD^+^‐dependent LDH [5]. Oxidation of ethanol possesses the same energetic problem as oxidation of lactate. The standard redox potential of ethanol/acetaldehyde pair is also −190 mV. In *A. woodii*, the energetic hurdle is overcome by a bifunctional alcohol/acetaldehyde dehydrogenase, AdhE. The enzyme apparently lowers the acetaldehyde concentration by oxidation to acetyl‐CoA thus making ethanol‐dependent NAD^+^ reduction feasible [20]. The same principle is observed in *M. thermoacetica* for lactate oxidation but most likely catalyzed by two subsequent enzymes: the LDH and EtfABCX complex. The latter bifurcates electrons to menaquinone (MQ) and NAD^+^. The cytochrome‐ and quinone‐containing *M. thermoacetica* can lower the NADH concentration by coupling NADH oxidation to reduction of menaquinone (MQ, *E*_0_′ = −74 mV). Moreover, this reaction kills two birds with one stone: not only the NADH concentration is lowered which makes lactate‐dependent NAD^+^ reduction energetically feasible but in addition, the exergonic NADH‐dependent MQ reduction drives the endergonic reduction of ferredoxin by electron bifurcation. Reoxidation of reduced ferredoxin as well as of menaquinol (MQH_2_) provides additional ATP by electron transport phosphorylation. Thus, a NAD^+^‐dependent LDH connected with an electron‐bifurcating EtfABCX complex is energetically advantageous. As suggested earlier, MQH_2_ is assumed to be the electron donor for the reduction of methylene‐THF (*E*_0_′ = −200 mV) [6]. This endergonic reaction is again driven by electron confurcation with NADH as electron donor (Fig. 5). Acetogenesis from lactate involving the electron‐bifurcating LDH in *A. woodii* gives only 0.25 mol of ATP/acetate, whereas acetate formation from lactate in *M. thermoacetica* gives 0.67 mol of ATP/acetate by substrate‐level phosphorylation plus additional ATP by electron transport phosphorylation with MQH_2_ and reduced ferredoxin as electron donors.

The determined temperature optimum of the LDH is in the range of temperature that supports growth of *M. thermoacetica*. The comparison to known NAD^+^‐dependent LDHs suggests that the LDH from *M. thermoacetica* is activated by FBP but we did not observe a stimulation. Unlike the tetrameric LDHs from *L. acidophilus*, *S. equinus*, *B. bifidum*, and *B. subtilis*, the LDH from *M. thermoacetica* is a dimeric protein [19, 21, 22, 23]. In contrast to many described LDHs, the optimal pH for the oxidation of lactate and reduction of pyruvate is identical (pH 8). Usually, the optimal pH for pyruvate reduction with NADH is at least one pH value lower, for, e.g., *B. subtilis* pyruvate reduction is optimal at pH 6.0 whereas the optimal pH for lactate oxidation is at pH 7.2 [19, 21]. The determined enzyme kinetics of the LDH from *M. thermoacetica* leads to the hypothesis that the favorable reaction is the formation of lactate by reducing pyruvate with NADH as reductant. The *K*_m_ for lactate (30 mm) and NAD^+^ (0.9 mm) of *B. subtilis* is similar to those from *M. thermoacetica* (lactate 20 mm and NAD^+^ 0.1 mm), whereas the *K*_m_ for lactate of the LDH from *L. acidophilus* is 360 mm [21, 23]. In contrast, the *K*_m_ values for pyruvate and NADH were 1.5 mm and 69 μm, respectively, reflecting the physiological role of the enzyme in lactate production [19, 23]. Similar *K*_m_ values for pyruvate (1 mm) and NADH (0.1 mm) were determined for the LDH from *M. thermoacetica*.

Thermophilic bacteria and their enzymes are of increasing interest in biotechnological applications due to multiple advantages including reduced cooling/heating cost, lower contamination risks, higher turnover rates, higher solubilization of lignose‐based feedstock, and higher gas–liquid mass transfer rates [24, 25]. The production of lactate from renewable resources or waste is a desirable biotechnological application, since lactate as precursor for, e.g., bioplastic production has an increasing demand. A key feature of acetogenic bacteria is the ability to utilize gaseous substrates such as the waste gases H_2_, CO_2_, and CO. The conversion of H_2_ + CO_2_ to lactate was already shown for the mesophilic acetogen *A. woodii* but not for thermophilic acetogens [26]. So far, lactate formation by *M. thermoacetica* was not observed. Currently, the bioenergetics of *M. thermoacetica* is not well understood, which makes it difficult to increase ATP yields by genetic modification. Member of the genus *Moorella* are, so far, the only thermophilic acetogenic bacteria with a LDH. *T. kivui*, also a thermophilic acetogenic bacterium, cannot produce lactate due to a lack of LDH [27, 28, 29]. *T. kivui* can be genetically modified and might be a suitable platform for using the LDH from *M. thermoacetica* to produce lactate from waste gas [30].

For this purpose, a LDH is required which shows high activity at 66 °C, the optimal growth temperature of *T. kivui*. There are a just few thermophilic LDHs described including the LDH from *T. maritima*, *B. caldolyticus*, and *G. stearothermophilus*. When comparing the LDH from *M. thermoacetica* to the LDHs from *T. maritima*, *B. caldolyticus*, and *G. stearothermophilus*, the advantages of using the LDH from *M. thermoacetica* become clear (Table 2) [31, 32, 33]. The LDH from *M. thermoacetica* has its temperature optimum at 65 °C whereas the optimum for the LDH from *T. maritima*, *B. caldolyticus*, and *G. stearothermophilus* is at 55 °C, 63 °C, and 55 °C, respectively [23, 24, 25, 26]. The LDH from *B. caldolyticus* has its optimum at 63 °C but neither the *V*_max_ of pyruvate reduction (40–120 U·mg^−1^) nor the *K*_m_ value for pyruvate (6–15 mm) are as good as the values for the LDH from *M. thermoacetica*. The LDH from *G. stearothermophilus* has striking high activities with up to 2500 U·mg^−1^ at 55 °C but the *K*_m_ for NADH is 2 times higher compared to the LDH from *M. thermoacetica* [33]. On the other hand, when using lactate as feedstock, the LDH from *M. thermoacetica* has the highest affinity towards lactate with 20 mm and a *V*_max_ of 60 U·mg^−1^. The *K*_m_ for lactate from *T. maritima*, *B. caldolyticus*, and *G. stearothermophilus* are either higher (up to 400 mm) or have not determined [31, 32, 33]. Under this aspect, the LDH from *M. thermoacetica* is the favorable thermophilic enzyme for the conversion of lactate to pyruvate.

**Table 2:** Comparison of thermophilic LDHs from *Moorella thermoacetica*, *Thermotoga maritima*, *Bacillus caldolyticus*, and *Geobacillus stearothermophilus.* n.d., not determined, ?, postulated FBP dependency.

Characteristics	*M. thermoacetica*	*T. maritima*	*B. caldolyticus*	*G. stearothermophilus*
*V*_max_ lactate oxidation	60 U·mg^−1^	n.d.	n.d	n.d.
*K*_m_ lactate	20 mm	25 mm	n.d.	400 mm
*K*_m_ NAD^+^	0.5 mm	0.09 mm	n.d.	0.15 mm
*V*_max_ pyruvate reduction	237 U·mg^−1^	800 U·mg^−1^	40–120 U·mg^−1^	1500 U·mg^−1^ (−FBP) 2500 U·mg^−1^ (+FBP)
*K*_m_ pyruvate	1 mm	0.06 mm	6–15 mm	6.6 mm (−FBP) 0.4 mm (+FBP)
*K*_m_ NADH	0.1 mm	0.03 mm	0.11–0.14 mm	0.24 mm
Temp. optimum	65 °C	55 °C	63 °C	55 °C pyruvate 65 °C lactate
pH optimum lactate oxidation	8.0	7.5	n.d.	8.5
pH optimum pyruvate reduction	8.0	6.0	6.2	6.0
Stereospecificity	l‐lactate	l‐lactate	n.d.	l‐lactate
FBP dependency	+/?	+	n.d.	+

**Fig. 6:**
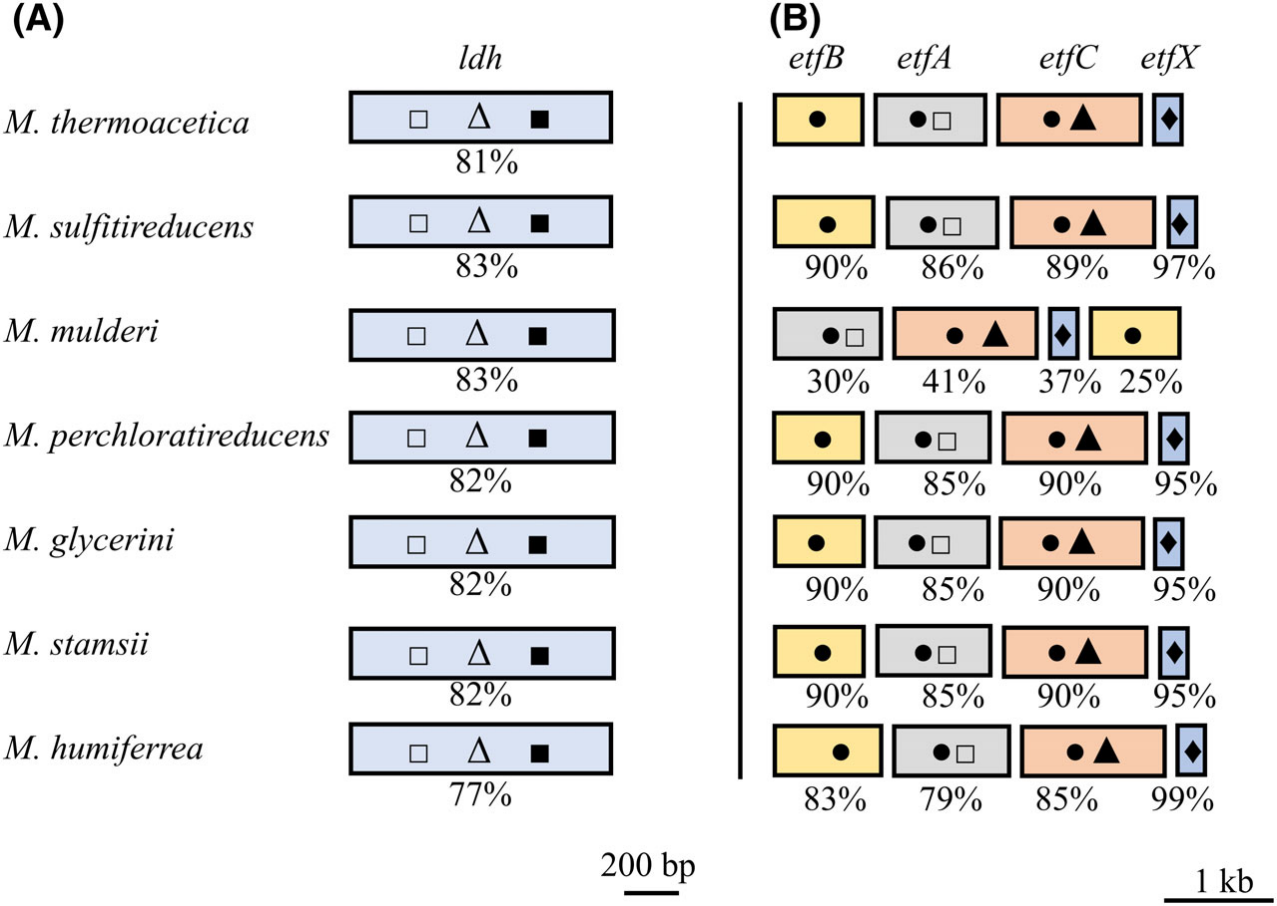
Comparison of LDHs and EtfABCX complexes in different *Moorella* strains. The LDH from *Moorella thermoacetica* is compared to enzymes from different *Moorella* strains (A). Similarity is given in %. □, Rossmann fold NAD(P)^+^ binding domain; Δ, fructose‐1,6‐bisphosphate binding domain; ■, active site. The EtfABCX complex from *M. thermoacetica* and different *Moorella* strains is compared (B). Same color indicates similar genes, similarity is given in %; ●, FAD binding domain; □, Rossmann fold NAD(P)^+^ binding domain; ▲, quinone binding domain; ♦, 4Fe‐4S binding domain.

Lactate oxidation is not only observed in *M. thermoacetica* but also in other *Moorella* strains. Searching the genome of other strains revealed that the lactate dehydrogenase is present in all *M. thermoacetica* strains and also in the *Moorella* strains *M. sulfitireducens*, *M. mulderi*, *M. perchloratireducens*, *M. glycerini*, *M. stamsii*, and *M. humiferrea* with a similarity of 81%, 83%, 82%, 82%, 82%, and 77%, respectively (Fig. 6A). The EtfABCX complex is suggested to be essential for growth on lactate. Therefore, we searched and found genes encoding this electron‐bifurcating complex in the strains mentioned above, only in *M. mulderi* the order of *etfABCX* encoding genes is different (Fig. 6B). Based on these analyses, it can be suggested that those strains can grow on lactate, but this is contradicting the literature, since *M. stamsii* and *M. perchloratireducens* are reported to be unable to utilize lactate [34, 35, 36, 37, 38, 39].

Although the biochemical characteristics of the LDH has been reported in this work, the biochemical characterization of the EtfABCX complex should be next, to outline its potential role in the enzymology of the lactate metabolism in *M. thermoacetica* and beyond. This might also give insights into the quinone pool reduction, an essential, but unknown physiological processes in *Moorella* species. The quinone pool might be involved in processes including reduction of alternative electron acceptors such as DMSO or nitrate, as second electron donor for the putative electron‐bifurcating methylene‐THF reductase or as electron donor for the cytochrome *bd*‐oxidase (Fig. 5) [40, 41, 42, 43].

## Conflict of interest

The authors declare no conflict of interest.

## Author contributions

FPR and VM designed the experiments. FPR performed the experiments. FPR and VM wrote the paper.

## Supporting information


**Fig. S1.** Sequence alignment of LDH. LDH from *M. thermoacetica* is compared to the NAD^+^‐dependent LDHs from *Lactobacillus acidophilus*, *Streptococcus equinus*, *Bifidobacterium bifidum*, *Bacillus subtilis*, *Thermotoga maritima*, *Bacillus caldolyticus* and *Geobacillus stearothermophilus*. Yellow, Rossmann fold; red, substrate binding domain; blue, fructose‐1,6‐bisphosphate binding domain; green, active site. *, identical subunit; :, conserved substitution; .; semi‐conserved substitution.

## Data Availability

All other data of this study are available from the corresponding author upon reasonable request.

## References

[1] Drake HL , Gößner AS and Daniel SL (2008) Old acetogens, new light. Ann N Y Acad Sci 1125, 100–128. 18378590 10.1196/annals.1419.016

[2] Bennett BD , Kimball EH , Gao M , Osterhout R , van Dien SJ and Rabinowitz JD (2009) Absolute metabolite concentrations and implied enzyme active site occupancy in *Escherichia coli* . Nat Chem Biol 5, 593–599. 19561621 10.1038/nchembio.186 PMC2754216

[3] Katsyv A , Essig M , Bedendi G , Sahin S , Milton RD and Müller V (2023) Characterization of ferredoxins from the thermophilic, acetogenic bacterium *Thermoanaerobacter kivui* . FEBS J 290, 4107–4125. 37074156 10.1111/febs.16801

[4] Ljungdahl LG (1986) The autotrophic pathway of acetate synthesis in acetogenic bacteria. Annu Rev Microbiol 40, 415–450. 3096193 10.1146/annurev.mi.40.100186.002215

[5] Weghoff MC , Bertsch J and Müller V (2015) A novel mode of lactate metabolism in strictly anaerobic bacteria. Environ Microbiol 17, 670–677. 24762045 10.1111/1462-2920.12493

[6] Rosenbaum FP , Poehlein A , Egelkamp R , Daniel R , Harder S , Schlüter H and Schoelmerich MC (2021) Lactate metabolism in strictly anaerobic microorganisms with a soluble NAD^+^‐dependent L‐lactate dehydrogenase. Environ Microbiol 23, 4661–4672. 34190373 10.1111/1462-2920.15657

[7] Katsyv A , Schoelmerich MC , Basen M and Müller V (2021) The pyruvate:ferredoxin oxidoreductase of the thermophilic acetogen, *Thermoanaerobacter kivui* . FEBS Open Bio 11, 1332–1342. 10.1002/2211-5463.13136 PMC8091585 33660937

[8] Thauer RK , Jungermann K and Decker K (1977) Energy conservation in chemotrophic anaerobic bacteria. Bacteriol Rev 41, 100–180. 860983 10.1128/br.41.1.100-180.1977 PMC413997

[9] Futai M and Kimura H (1977) Inducible membrane‐bound L‐lactate dehydrogenase from *Escherichia coli*. Purification and properties. J Biol Chem 252, 5820–5827. 18473

[10] de Albuquerque TL , Marques Junior JE , de Queiroz LP , Ricardo ADS and Rocha MVP (2021) Polylactic acid production from biotechnological routes: a review. Int J Biol Macromol 186, 933–951. 34273343 10.1016/j.ijbiomac.2021.07.074

[11] Hungate RE (1969) A roll tube method for cultivation of strict anaerobes. In Methods in Microbiology ( Norris JR and Ribbons DW , eds), pp. 117–132. Academic Press, New York, NY and London.

[12] Bryant MP (1972) Commentary on the Hungate technique for culture of anaerobic bacteria. Am J Clin Nutr 25, 1324–1328. 4565349 10.1093/ajcn/25.12.1324

[13] Bradford MM (1976) A rapid and sensitive method for the quantification of microgram quantities of protein utilizing the principle of proteine‐dye‐binding. Anal Biochem 72, 248–254. 942051 10.1016/0003-2697(76)90527-3

[14] Laemmli UK (1970) Cleavage of structural proteins during the assembly of the head of bacteriophage T4. Nature 227, 680–685. 5432063 10.1038/227680a0

[15] Schönheit P , Wäscher C and Thauer RK (1978) A rapid procedure for the purification of ferredoxin from *Clostridia* using polyethylenimine. FEBS Lett 89, 219–222. 658409 10.1016/0014-5793(78)80221-x

[16] Jumper J , Evans R , Pritzel A , Green T , Figurnov M , Ronneberger O , Tunyasuvunakool K , Bates R , Žídek A , Potapenko A *et al*. (2021) Highly accurate protein structure prediction with AlphaFold. Nature 596, 583–589. 34265844 10.1038/s41586-021-03819-2 PMC8371605

[17] Mirdita M , Schutze K , Moriwaki Y , Heo L , Ovchinnikov S and Steinegger M (2022) ColabFold: making protein folding accessible to all. Nat Methods 19, 679–682. 35637307 10.1038/s41592-022-01488-1 PMC9184281

[18] Goddard TD , Huang CC , Meng EC , Pettersen EF , Couch GS , Morris JH and Ferrin TE (2018) UCSF ChimeraX: meeting modern challenges in visualization and analysis. Protein Sci 27, 14–25. 28710774 10.1002/pro.3235 PMC5734306

[19] Garvie EI (1980) Bacterial lactate dehydrogenases. Microbiol Rev 44, 106–139. 6997721 10.1128/mr.44.1.106-139.1980 PMC373236

[20] Bertsch J , Siemund AL , Kremp F and Müller V (2016) A novel route for ethanol oxidation in the acetogenic bacterium *Acetobacterium woodii*: the acetaldehyde/ethanol dehydrogenase pathway. Environ Microbiol 18, 2913–2922. 26472176 10.1111/1462-2920.13082

[21] Yoshida A and Freese E (1975) Lactate dehydrogenase from *Bacillus subtilis* . Methods Enzymol 41, 304–309. 236452 10.1016/s0076-6879(75)41069-2

[22] Hensel R , Mayr U , Fujiki H and Kandler O (1977) Comparative studies of lactate dehydrogenases in lactic acid bacteria. Amino‐acid composition of an active‐site region and chemical properties of the L‐lactate dehydrogenase of *Lactobacillus casei*, *Lactobacillus curvatus*, *Lactobacillus plantarum*, and *Lactobacillus acidophilus* . Eur J Biochem 80, 83–92. 411654 10.1111/j.1432-1033.1977.tb11859.x

[23] Gasser F , Doudoroff M and Contopoulos R (1970) Purification and properties of NAD‐dependent lactic dehydrogenases of different species of *Lactobacillus* . J Gen Microbiol 62, 241–250. 4321865 10.1099/00221287-62-2-241

[24] Cowan DA , Albers SV , Antranikian G , Atomi H , Averhoff B , Basen M , Driessen AJM , Jebbar M , Kelman Z , Kerou M *et al*. (2024) Extremophiles in a changing world. Extremophiles 28, 26. 38683238 10.1007/s00792-024-01341-7 PMC11058618

[25] Vavitsas K , Glekas PD and Hatzinikolaou DG (2022) Synthetic biology of thermophiles: taking bioengineering to the extremes? Appl Microbiol 2, 165–174.

[26] Moon J , Waschinger LM and Müller V (2023) Lactate formation from fructose or C1 compounds in the acetogen *Acetobacterium woodii* by metabolic engineering. Appl Microbiol Biotechnol 107, 5491–5502. 37417977 10.1007/s00253-023-12637-7 PMC10390620

[27] Hess V , Poehlein A , Weghoff MC , Daniel R and Müller V (2014) A genome‐guided analysis of energy conservation in the thermophilic, cytochrome‐free acetogenic bacterium *Thermoanaerobacter kivui* . BMC Genomics 15, 1139. 25523312 10.1186/1471-2164-15-1139 PMC4320612

[28] Katsyv A , Jain S , Basen M and Müller V (2021) Electron carriers involved in autotrophic and heterotrophic acetogenesis in the thermophilic bacterium *Thermoanaerobacter kivui* . Extremophiles 25, 513–526. 34647163 10.1007/s00792-021-01247-8 PMC8578170

[29] Daniel SL , Hsu T , Dean SI and Drake HL (1990) Characterization of the H_2_‐dependent and CO‐dependent chemolithotrophic potentials of the acetogens *Clostridium thermoaceticum* and *Acetogenium kivui* . J Bacteriol 172, 4464–4471. 2376565 10.1128/jb.172.8.4464-4471.1990 PMC213276

[30] Basen M , Geiger I , Henke L and Müller V (2018) A genetic system for the thermophilic acetogenic bacterium *Thermoanaerobacter kivui* . Appl Environ Microbiol 84, e02210‐17. 29150512 10.1128/AEM.02210-17 PMC5772241

[31] Weerkamp A and Mac Elroy RD (1972) Lactate dehydrogenase from an extremely thermophilic *Bacillus* . Arch Mikrobiol 85, 113–122. 4342162 10.1007/BF00409292

[32] Wrba A , Jaenicke R , Huber R and Stetter KO (1990) Lactate dehydrogenase from the extreme thermophile *Thermotoga maritima* . Eur J Biochem 188, 195–201. 2318202 10.1111/j.1432-1033.1990.tb15388.x

[33] Schar HP and Zuber H (1979) Structure and function of L‐lactate dehydrogenases from thermophilic and mesophilic bacteria. (I) Isolation and characterization of lactate dehydrogenases from thermophilic and mesophilic bacilli. Hoppe Seylers Z Physiol Chem 360, 795–807. 114469 10.1515/bchm2.1979.360.2.795

[34] Alves JI , van Gelder AH , Alves MM , Sousa DZ and Plugge CM (2013) *Moorella stamsii* sp. nov., a new anaerobic thermophilic hydrogenogenic carboxydotroph isolated from digester sludge. Int J Syst Evol Microbiol 63, 4072–4076. 23749275 10.1099/ijs.0.050369-0

[35] Balk M , van Gelder T , Weelink SA and Stams AJ (2008) (Per)chlorate reduction by the thermophilic bacterium *Moorella perchloratireducens* sp. nov., isolated from underground gas storage. Appl Environ Microbiol 74, 403–409. 17981952 10.1128/AEM.01743-07 PMC2223267

[36] Nepomnyashchaya YN , Slobodkina GB , Baslerov RV , Chernyh NA , Bonch‐Osmolovskaya EA , Netrusov AI and Slobodkin AI (2012) *Moorella humiferrea* sp. nov., a thermophilic, anaerobic bacterium capable of growth via electron shuttling between humic acid and Fe(III). Int J Syst Evol Microbiol 62, 613–617. 21531740 10.1099/ijs.0.029009-0

[37] Balk M , Weijma J , Friedrich MW and Stams AJ (2003) Methanol utilization by a novel thermophilic homoacetogenic bacterium, *Moorella mulderi* sp. nov., isolated from a bioreactor. Arch Microbiol 179, 315–320. 12637975 10.1007/s00203-003-0523-x

[38] Slobodkin A , Reysenbach AL , Mayer F and Wiegel J (1997) Isolation and characterization of the homoacetogenic thermophilic bacterium *Moorella glycerini* sp. nov. Int J Syst Bacteriol 47, 969–974. 9336894 10.1099/00207713-47-4-969

[39] Slobodkina GB , Merkel AY , Kuchierskaya AA and Slobodkin AI (2022) *Moorella sulfitireducens* sp. nov., a thermophilic anaerobic bacterium isolated from a terrestrial thermal spring. Extremophiles 26, 33. 36352059 10.1007/s00792-022-01285-w

[40] Rosenbaum FP and Müller V (2021) Energy conservation under extreme energy limitation: the role of cytochromes and quinones in acetogenic bacteria. Extremophiles 25, 413–424. 34480656 10.1007/s00792-021-01241-0 PMC8578096

[41] Rosenbaum FP , Poehlein A , Daniel R and Müller V (2022) Energy‐conserving dimethyl sulfoxide reduction in the acetogenic bacterium *Moorella thermoacetica* . Environ Microbiol 24, 2000–2012. 35278024 10.1111/1462-2920.15971

[42] Das A , Silaghi‐Dumitrescu R , Ljungdahl LG and Kurtz DM Jr (2005) Cytochrome *bd* oxidase, oxidative stress, and dioxygen tolerance of the strictly anaerobic bacterium *Moorella thermoacetica* . J Bacteriol 187, 2020–2029. 15743950 10.1128/JB.187.6.2020-2029.2005 PMC1064043

[43] Das A , Hugenholtz J , Van Halbeek H and Ljungdahl LG (1989) Structure and function of a menaquinone involved in electron transport in membranes of *Clostridium thermoautotrophicum* and *Clostridium thermoaceticum* . J Bacteriol 171, 5823–5829. 2808299 10.1128/jb.171.11.5823-5829.1989 PMC210442

