# Supplementary material for: Purification and characterization of a thermophilic NAD +‐dependent lactate dehydrogenase from Moorella thermoacetica
Source: FEBS Open Bio. 2025 Jan 13;15(5):714–25. doi: 10.1002/2211-5463.13964 (PMC12051018; doi:10.1002/2211-5463.13964)
Supplement: Supplementary file 1 — Fig. S1. Sequence alignment of LDH. LDH from M. thermoacetica is compared to the NAD+‐dependent LDHs from Lactobacillus acidophilus, Streptococcus equinus, Bifidobacterium bifidum, Bacillus subtilis, Thermotoga maritima, Bacillus caldolyticus and Geobacillus stearothermophilus. Yellow, Rossmann fold; red, substrate binding domain; blue, fructose‐1,6‐bisphosphate binding domain; green, active site. *, identical subunit; :, conserved substitution; .; semi‐conserved substitution. [file FEB4-15-714-s001.docx]

**Supplementary Information**

for the manuscript:

**Purification and characterization of a thermophilic NAD^+^-dependent lactate dehydrogenase from *Moorella thermoacetica***

**Florian P. Rosenbaum and Volker Müller***

Department of Molecular Microbiology & Bioenergetics, Institute of Molecular Biosciences, Johann Wolfgang Goethe University, Frankfurt am Main, Germany

^*^correspondence address: Prof. Volker Müller, Department of Molecular Microbiology & Bioenergetics, Institute of Molecular Biosciences, Johann Wolfgang Goethe University, Max-von-Laue-Str. 9, 60438, Frankfurt am Main, Germany; Phone: 49-6979829507; Fax: 49-69-79829306;

E-mail: [vmueller@bio.uni-frankfurt.de](mailto:vmueller@bio.uni-frankfurt.de)

**Fig. S1**

**Supplementary figures**

**Fig. S1. Sequence alignment of LDH.** LDH from *M. thermoacetica* is compared to the NAD^+^-dependent LDHs from *Lactobacillus acidophilus*, *Streptococcus equinus*, *Bifidobacterium bifidum*, *Bacillus subtilis*, *Thermotoga maritima, Bacillus caldolyticus* and *Geobacillus stearothermophilus.* Yellow, Rossmann fold; red, substrate binding domain; blue, fructose-1,6-bisphosphate binding domain; green, active site. *, identical subunit; :, conserved substitution; .; semi-conserved substitution.
